# “Tumor immunology meets oncology (TIMO) XV”, April 25th–27th 2019, Halle/Saale, Germany

**DOI:** 10.1007/s00262-020-02491-1

**Published:** 2020-02-05

**Authors:** André Steven, Chiara Massa, Barbara Seliger

**Affiliations:** Institute of Medical Immunology, Martin-Luther-University Halle-Wittenberg, Magdeburger Str. 2, 06112 Halle, Germany

**Keywords:** TIMO 2019, Immune escape, Tumor microenvironment, Immunomonitoring immunotherapy, Immune signatures

## Abstract

Novel insights into basic and translational tumor immunology including immunotherapies were presented by national and international scientists and clinicians at the TIMO XV meeting in Halle.

## Introduction

The symposium “Tumor immunology meets oncology XV (TIMO XV)” was held from the 25th until April 27th, 2019 at the Medical Faculty of the Martin Luther University Halle-Wittenberg in Halle, Germany. There were more than 130 participants from 12 different nations including 20 speakers at the symposium, 19 speakers at the workshop and 15 poster presenters. The workshop consisted of short talks and posters from young scientists or companies presenting their novel technologies and/or data from clinical trials. The topics covered by the speakers ranged from basic to translational science and clinical phase II and III trials. These include immune escape and resistance mechanisms of tumors and strategies leading to their reversion, the cellular composition of the tumor infiltrate, tumor mutational burden (TMB) and its role for anti-tumoral immune responses as well as its use as predictive biomarker, function of natural killer (NK) and T cells and epidemiologic studies up to clinical trials using adoptive cell therapy (ACT), epigenetic drugs and immune checkpoint inhibitors (iCPI) alone or in combination.

**Arndt Hartmann** (Institute of Pathology, University Hospital Erlangen, Erlangen, Germany) presented different problems that a clinician has to affront treating bladder cancer patients. First, he presented data supporting a new RNA expression-based consensus classification of muscle-invasive bladder cancer, which incorporates an immune contexture classification and identifies six cancer subtypes based on the presence of inflammation, which correlate with the clinical outcome of patients. Next, he addressed the problem of patients’ stratification for immunotherapy. Indeed for each approved drug targeting the programmed death receptor (PD)1/programed death ligand 1 (PD-L1) axis, namely, Atezolizumab, Pembrolizumab, Nivolumab and Durvalumab, a different screening assay is suggested by the Food and Drug Administration (FDA) and European Medical Agency (EMA) that not only use different antibodies (Abs) for staining PD-L1 (SP142, 22c3, 28-8 and SP263, respectively), but also different cut off values, which take into consideration membranous/cytoplasmic staining of tumor and/or immune cells. Since the different Abs have a similar “efficacy” in staining immune cells, but a distinct staining pattern for tumor cells with, e.g., a reduced capacity of tumor cell staining for SP142, the classification of a patient as PD-L1 positive or negative, and thus the eligibility for iCPI therapy highly relay on the performed staining. A similar problem with cut off levels is found for TMB. In many tumor types, higher levels of TMB correlate with response to immunotherapy, but there exists no clear cut off value.

**Marc Schmitz** (Institute of Immunology, Dresden, Germany) evaluated the immune architecture in another urologic tumor, renal cell carcinoma (RCC), focusing on monocytes expressing the 6-sulfo LacNAc (slan) modification of PSGL1, shortly termed slanMo. Increased frequencies of slanMos were detected in RCC tissues compared to tumor-free tissues. slanMos were also found in the majority of metastatic lymph nodes and distant metastases from RCC patients. A higher density of slanMos was significantly associated with a reduced progression-free and overall survival of RCC patients. Tumor-infiltrating slanMos displayed a tolerogenic phenotype characterized by low expression of costimulatory molecules and high expression levels of interleukin (IL)-10. Further studies revealed that RCC cells markedly inhibit slanMo-induced T-cell proliferation and programming as well as natural killer cell activation. Subsequently, he presented data on the impact of neoadjuvant radio-chemotherapy (nRCT) on the frequency and phenotype of rectal cancer-infiltrating plasmacytoid dendritic cells (pDCs) and CD8^+^ T cells. A significantly higher density of pDCs in comparison to pre-nRCT tissue samples was found. nRCT also increased the percentage of pDCs expressing the maturation marker CD83 and the pro-inflammatory cytokine interferon (IFN)-α. Further studies revealed that nRCT significantly enhances the proportion of rectal cancer-infiltrating CD8^+^ T cells expressing the cytotoxic effector molecule granzyme B. These findings indicate that nRCT significantly alters the frequency and phenotype of pDCs and CD8^+^ T cells, which may influence the clinical response of rectal cancer patients to nRCT.

**Barbara Seliger** (Institute of Medical Immunology, Martin Luther University Halle-Wittenberg, Halle, Germany) presented data on the different escape mechanisms used by tumor cells focusing mostly on changes in the major histocompatibility complex (MHC) class I antigen processing machinery (APM). Evaluation of biopsies from oral squamous cell carcinoma (OSCC) patients highlighted a significant downregulation of different APM components in malignant versus non-malignant cells. Further evaluation indicated a correlation of higher levels of beta2-microglobulin (β2-m) in stage 1 and 2 OSCC with a lower overall survival (OS). Deeper histological evaluation linked the level of HLA class I positivity to the density of CD4^+^ and CD8^+^ T-cell infiltrate and inversely with the presence of suppressive Foxp3^+^ or PD-L1^+^ cells near the CD8^+^ T cells. Similar data were obtained by in silico analysis of melanoma using The Cancer Genome Atlas (TCGA) demonstrating a correlation between low APM component expression levels and reduced patients’ survival. Furthermore, the loss or decreased APM component expression during ACT directly correlated with resistance development to this treatment. Characterisation of the mechanism(s) leading to a down regulation of APM and/or HLA molecules resulted in the identification of posttranscriptional processes. Different microRNAs (miRNAs), including miR-200a, could bind to the 3′ untranslated region (UTR) of the transporter associated with antigen processing (TAP)1 and affect TAP1 and consequently human leukocyte antigen (HLA) class I surface expression when transfected in different melanoma cell lines. In addition, miRNAs regulating the expression of HLA-G alone or targeting also classical HLA class I molecules were detected. Finally, the role of the extracellular matrix (ECM) protein biglycan (BGN) on MHC class I expression was provided. BGN expression was downregulated upon oncogenic transformation and its reconstitution in malignant cells was able to upregulate MHC class I surface expression thereby increasing CD8^+^ T-cell infiltration in vivo. Mechanistically, BGN reduces the amount of miR-21, which binds to the 3′UTR of TAP1 leading to recover of the MHC class I expression. Clinically, such feedback loop can be of relevance, since TCGA data analysis of mammary carcinoma highlighted an increased survival for patients with high levels of HLA-B and BGN and low levels of miR-21. These novel data with an involvement of ECM components suggest a more complex control of MHC class I APM components in tumor cells.

**Ping-Chih Ho** (Lausanne, Switzerland) proposed a new therapeutic strategy based on changes in the metabolism to selectively inhibit regulatory T cells (Tregs). Indeed, a comparison between circulating and tumor-infiltrating Tregs highlights differences in the expression of many metabolic pathways. Flow cytometric analysis confirmed an enhanced lipid uptake and content in Tregs that is associated with higher levels of CD36 and the fatty acid binding proteins − 4 and − 5. Based on this observation, the role of CD36 in Treg function was determined using a FoxP3-specific CD36 knock out (k.o.) mouse model. In these mice, injected tumors were smaller and had a reduced Treg infiltration and when compared to circulating Tregs had a lower lipid content, lower expression of GITR and OX40 and were overall less functional resulting in a higher infiltration by CD4^+^ and CD8^+^ T cells that could also produce IFN-γ and tumor necrosis factor (TNF)-α. The infiltrating Tregs from CD36 k.o. mice had fewer mitochondria with abnormal crista. These data suggest an impaired OXPHOS rendering the cells sensitive to the low pH and high lactic acid concentration in the tumor microenvironment (TME). Indeed, Tregs have high levels of lactate dehydrogenase (LDH) B that can reconvert lactate to pyruvate, which requires a functional NAD–NADH system based on the mitochondria complex I. Preliminary evaluation of the therapeutic applicability of such discovery was performed by treating tumor bearing mice with a blocking anti-CD36 Ab leading to a synergistic effect in combination with an anti-PD1 Ab.

**Andreas Beilhack** (University of Würzburg, Germany) presented data for the opposite purpose, such as promoting Treg function to reduce the graft-versus-host disease (GvHD) that frequently occurs in leukemia patients undergoing hematopoietic cell transplantation. To this aim, he explored the differential expression patterns and functions of tumor necrosis factor receptors (TNF-R). The type I TNF-R recognizes both soluble and membrane bound TNF-α, whereas the type II TNF-R requires oligomerization and can thus transduce only in response to membrane bound/oligomeric TNF signals. Stimulating TNF-R2 activates natural arising (n)Tregs while inhibiting induced (i)Tregs. His team developed STAR2, a selective agonist for TNF-RII composed of three TNF trimers bearing point mutations that abolish the interaction with TNF-RI. Treatment of mice undergoing allogeneic hematopoietic cell transplantation with STAR2 resulted in an amplification of recipient nTregs resulting in lower levels of GvHD while preserving the desired immunological graft-versus-leukemia effect and protection from opportunistic infections.

**Pamela Ohashi** (University of Toronto, Toronto, Canada) initially presented data on different phenotypes of Tregs infiltrating ovarian cancer and melanoma that could also be responsible for the distinct responses of the two tumor types to PD1 blockade. Indeed, Tregs infiltrating ovarian carcinoma express PD1 and also high levels of the inducible T-cell costimulator (ICOS). Double staining and expression profiling of the sorted population suggested that the PD1^int^ ICOS^hi^ cells are Tregs, which were confirmed by functional suppression assays, the presence of a demethylated FoxP3 promoter, and an activated phenotype characterized by the expression of cytotoxic T lymphocyte associated protein-4 (CTLA-4), OX40 and T-cell immunoglobulin and immune receptor tyrosine-based inhibitory motif (TIGIT). In comparison, Tregs infiltrating melanoma exhibit lower levels of PD1, lack ICOS expression and have also lower expression of FoxP3 suggesting a less active phenotype.

Next to this, P. Ohashi presented studies evaluating the properties of murine T cells polarized to the Tc1, Tc2, Tc9, Tc17 and Tc22 lineages. The different T-cell subsets were then analyzed for their cytokine secretion pattern, expression of activation markers and cytotoxic activity both in vitro and upon transfer into tumor bearing mice. While Tc1 and Tc22 displayed a similar cytotoxicity in vitro, the Tc22 was superior in inducing tumor regression in vivo. Importantly, this Tc22 population was found in tumor infiltrating lymphocyte (TILs) from ovarian cancer patients, and correlated with recurrence free survival.

**Shuji Ogino** (Harvard Medical School, Boston, USA) described that any disease state including neoplasms represents fundamentally heterogeneous pathological processes due to cellular and molecular alterations and influences of the exposome (including the microbiome) and the immune system. However, most traditional research approaches have not examined influences of the exposome on tumor-immune interactions. To address this, the integrative scientific field of molecular pathological epidemiology (MPE) can investigate influences of the exposome (microbial, dietary, lifestyle, environmental, pharmacological, and other exposures) on tumor-immune interactions, thereby informing immunoprevention and immunotherapy research. Using over 1500 colorectal cancer (CRC) cases with rich data on immune response, whole exome sequencing of tumor and normal DNA, tumor neoantigens, tissue microorganisms (e.g., *Fusobacterium nucleatum*), and clinical outcomes, their proof-of-principle microbiology–immunology–MPE studies have shown that several cancer risk factors appear to influence carcinogenic processes through their effects on tumor–microbial–immune interactions. These new research paradigms can provide possible paths for precision prevention and therapy.

**Ofer Mandelboim** (Hadassah Medical School, Jerusalem, Israel) represent further data on the negative effect of *Fusobacterium nucleatum* by evaluating its interaction with NK cells. Indeed, these bacteria can directly bind to NK cells. Using a ligand screening, the Fap2 protein of the bacteria has been shown to bind to TIGIT, an inhibitory Ig-receptor expressed on all NK cells as well as on the majority of T cells thereby blocking their cytotoxic activity. Moreover, the Fap2 protein is responsible for the enrichment of the bacteria in the tumor, since it binds also to Gal–Gal–Nac that is overexpressed in CRCs. Thus, when the bacterium normally present in the saliva reach the blood stream, it can accumulate within the tumor and impair the cytotoxic activity of infiltrating NK cells. Therefore, it is hypothesed that the “tumor homing” property of Fap2/*Fusobacterium nucleatum* could be hijacked for therapeutic approaches aiming at targeting specific compounds to the tumor site.

Further work on TIGIT resulted in the identification of a new ligand, namely, Nectin-4, which in contrast to the previously known TIGIT ligands PVR, nectin-2 and -3 binding in addition to the inhibitory receptor CD112R and/or to the activating receptor DNAM1, only bind to TIGIT. To evaluate the therapeutic potential of targeting Nectin4 to unleash NK-cell cytotoxicity, preliminary experiments in SCID mice transferred with human NK cells were implemented, since the murine TIGIT does not bind to murine Nectin-4. In such a setting, cells overexpressing Nectin-4 had enhanced tumor growth in the presence of NK cells. A blocking Ab against Nectin-4 could revert the phenotype in an NK-dependent way.

**Mathieu Bléry** (Innate Pharma, Marseille, France) demonstrated potential ways how to improve their functionality against cancer using NK cells. In particular, he presented the (i) unleashing and (ii) retargeting of NK cells as strategy. The first setting focused on NKG2A, an inhibitory receptor expressed on NK cells as well on some CD8^+^ T cells that upon recognition of its ligand HLA-E (Qa-1^b^ in mice) inhibits the cell effector functions. Preliminary experiments in the A20 lymphoma model, whose infiltrate contain NKG2A^+^ NK cells as well as PD1^+^ CD8^+^ T cells also co-expressing NKG2A, indicate that an anti-NKG2A Ab can improve response to PD1 blockade. Shifting to the human setting, many tumor types are positive for HLA-E. Head and neck squamous cell carcinoma (HNSCC) have an infiltrate containing NK as well as CD8^+^ T cells expressing NKG2A alone or co-expressing NKG2A and PD1 thus leading to clinical trials targeting both PD-L1 and NKG2A. Since NK cells are also responsible for the antibody-dependent cellular cytotoxicity (ADCC), unleashing of the NKG2A-mediated inhibition was also combined with Cetuximab treatment, an anti-epidermal growth factor receptor (EGF-R) Ab working mostly via ADCC, resulting in a 27.5% objective response rate (ORR) with one complete and ten partial responses in a phase II clinical trial (NCT02643550). The second approach consists in NK-cell engagers (NKCE), the equivalent for NK cells of bispecific Ab for T cells, but with three components. In addition to the Ab portion recognizing the tumor antigen of interest, in the presented cases CD20, and the Ab targeting the NK cells via the NKp46 receptor that differently from Nkp30, NKG2D or CD16 is retained in tumor infiltrating NK. There is also the Fc portion of the Ab that in its wild type form can bind to the CD16 receptor, thus providing a second recognition molecule for NK-cell targeting. To evaluate the role of this second binding motif for the functionality of the NKCE, the Fc portion has also been mutated to silence or enhance its binding to the CD16 receptor. In vitro and in vivo murine experiments indicate that the NKCE is able to induce tumor cell killing in an NK-dependent way and promote NK-cell infiltration of the tumor. Comparison of the different Fc moieties indicated that in vitro the Fc binding significantly enhance tumor cell killing and also in vivo there is a further reduction in tumor growth.

**Joost Kreijtz’** (Aduro Biotech Europe, Oss, Netherlands) focus was also on innate immunity, but on the phagocyte side. Many tumors upregulate the CD47 molecule that, upon binding to the signal regulatory protein α (SIRPα) on phagocytes, provides a “don’t eat me” signal to these cells. Challenges in targeting SIRPα come from the fact that the molecule belongs to a family with inhibitory as well as activating receptors. An alternative would be to target CD47, but it has been shown on multiple occasions that CD47 antibodies can induce anemia and thrombocytopenia in mice and cynomolgus monkeys due to the high expression of CD47 on erythrocytes. These findings were also reported in patients that were administered anti-CD47 Ab. Despite these challenges, various antibodies targeting SIRPα and CD47 are in development. Aduro has tested a mouse anti-SIRPα in combination with an anti-PD-1 in mice. This resulted in synergistic effects on tumor rejection also due to the enhanced cross talk between DCs and T cells promoted by the positive effect of SIRPα triggering on DCs. More importantly, Aduro developed ADU-1805, a pan-allele human SIRPα IgG2 antibody. ADU-1805 blocks CD47 binding to SIRPα and enhances the antibody-mediated tumor cell uptake by phagocytes. In contrast to CD47 Abs, ADU-1805 does not dampen the T-cell response in vitro. Safety assessment of ADU-1805 showed that it does not bind to red blood cells or platelets and thus does not suffer from the “sink effect” that CD47 Abs have. This was confirmed in the first toxicology study with ADU-1805 in cynomolgus monkeys. This study showed that the Ab was well tolerated and did not have an impact on the red blood cell compartment. Therefore, it is suggested that targeting innate immunity through SIRPα has great potential to enhance cancer immunotherapy.

**Dieter Kabelitz** (Institute of Immunology, Christian-Albrechts-University, Kiel, Germany) gave perspectives on the usage of gamma/delta (γδ) T cells in cancer immunotherapy. These cells are considered as a link between the innate and adaptive immune system, since they can recognize their target cells via the NKG2D molecule and the T-cell receptor (TCR), like NK and T cells, respectively. Unlike the αβ receptor, the γδ TCR is not as polymorphic and does not recognize peptides presented on HLA molecules, but respond to exogenous microbial substances like 4-hydroxy-3-methly-but-2-enyl-pyrophosphate (or HMBPP) as well as to endogenous pyrophosphates like isopentenyl-pyrophosphate (IPP), an intermediate product of the mevalonate pathway that accumulate in stressed and transformed neoplastic cells. These pyrophosphates bind to the intracellular domain of butyrophiline molecules causing a cytoskeleton-dependent change in their conformation that induces recognition by the γδ TCR and leads to activation and proliferation of the γδ T cells. Despite some report of a pro-tumorigenic activity of γδ T cells due to the IL-17-mediated expansion of myeloid-derived suppressor cells (MDSC) and neutrophils, the high proliferative capacity of γδ T cells as well as the favorable prognosis of cells bearing the Vγ9Vδ2 TCR in colorectal and prostate carcinoma promote their implementation for tumor immunotherapy. ACT of γδ T cells has been implemented in RCC, malignant melanoma (MM) and hepatocellular carcinoma (HCC) providing some positive results, but as for classical αβ T-cell-based ACT, there is the need to further optimize it. Proposed strategies consist for example in (i) usage of an Ab to favor the conformation changes in butyrophiline molecules to avoid usage of IPP, (ii) decreased immunosuppression by, e.g., inhibition of COX2, or (iii) by retargeting the tumor recognition via bispecific antibodies. Different strategies have been implemented like the tribody (Her2)2xVγ9 chain recognizing the HER-2/neu moiety and combining it with the one recognizing either the Vγ9 chain, to specifically targeting the γδ T cells, or the CD16 moiety that in addition to cytotoxic NK cells is also expressed on some γδ T cells belonging both to the TIL expressing the Vδ1 chain as well as the blood resident lymphocytes expressing the Vδ2 chain.

Furthermore, the effector functions of γδ T cells could be enhanced by cytokine treatment. For example, treatment of purified γδ T cells with transforming growth factor (TGF)-β induces IL-9 production as well as an upregulation of the adhesion molecules ICAM-1 or CD103 resulting in an enhanced capability to form the immunological synapse with target cells in vitro leading to an enhanced cytotoxicity.

**Gottfried Baier** (Innsbruck, Austria) presented another new target for tumor immunotherapy, namely, the orphan nuclear receptor NR2F6. Despite its activating ligand is unknown, the receptor is essential for transcription and NR2F6 k.o. mice display autoimmunity and high rejection of transplantable tumor. These effects are also evident in heterozygotes suggesting an important dosage role. Functionally, NR2F6 is bound to the DNA to stabilize the heterochromatin and avoid stochastic initiation of transcription and can be released only when the TCR is triggered for long time and with high affinity. Experiments in mice demonstrated that its loss does not affect the suppressive capability of Tregs, enhance the immune signature of CD8^+^ T cells resulting in stronger tumor rejection when anti-PD-L1 is provided. Shifting to the human setting, a mixed staining pattern of NR2F6 was found in infiltrating CD3^+^ T cells. Albeit it did not correlate with patients’ survival, NR2F6 protein expression was upregulated in more than 50% in TILs of non-small-cell lung carcinoma (NSCLC) patients. Notably, the number of NR2F6-expressing TILs was highly correlated to the abundance of PD1 and CTLA-4-expressing TILs in this patient cohort (*n* = 300). Preliminary evaluation of the siRNA-mediated downregulation of NR2F6 in CD4^+^ and CD8^+^ human primary T cells resulted in hyper-responsive cells even when the expression was reduced only to 50%.

Analysis of the T-cell repertoire in immunotherapy-treated melanoma patients was the topic of **Nathalie Labarrière** (French Institute of Health and Medical Research, Nantes, France) talk. Blood samples from HLA-A02^+^ melanoma patients undergoing PD1 blockade with Nivolumab Ab as a monotherapy were collected before and during treatment to evaluate changes in functionality and TCR repertoire of cells specific for the MelanA/Mart1 antigen. Characterization of Vβ chains of sorted cells highlighted that in all patients, there were substantial changes in MelanA-specific T-cell repertoire. Interestingly, new clonotypes were amplified in complete and partial responders. Phenotypically, these new clonotypes had a higher frequency of PD1^+^ cells, but were not functionally exhausted, since they still degranulated and secreted different cytokines. Furthermore, in the responder patients these new clonotypes had higher antigen avidity and expressed TIGIT frequently together with PD1. After 1 month of anti-PD1 Ab treatment, the percentage of double positive cells significantly correlated with the clinical benefit. To confirm that the frequency of double positive (PD1^+^ and TIGIT^+^) circulating T cells could be a marker of anti-PD1 efficiency, these T cells were deeply analyzed in a cohort of melanoma patients undergoing PD1 therapy. The frequency of this T-cell subset after 1 month of therapy was indeed increased in responding patients. With respect to single positive or double negative cells, the PD1^+^ TIGIT^+^ cells had higher expression levels of PD1, indicating a higher level of activation and higher frequencies of cells also co-expressing CD38 and HLA-DR. RNA sequencing highlighted that the double positive cells expressed higher levels of genes involved in proliferation, effector function and metabolism. Functionally, the highest numbers of antigen-responding cells were found in double positive cells, which were recorded after 1 month of therapy, suggesting that this circulating T-cell subset could be a relevant marker of anti-PD1 clinical efficacy.

**Joe Poh Sheng Yeong** (Department of Anatomical Pathology, Singapore General Hospital, Singapore) described the approaches used to identify biomarkers for the responsiveness to immunotherapy in the Asian population. From the methodological point of view, he presented their attempt to transform immunohistochemical characterization of patients’ tumors into a high throughput, automatable, affordable and not “too tissue consuming” approach using multispectral imaging (MSI). He presented different Ab panels combining up to six different markers that allow to deeply characterize the tumor infiltrate and the use of the SOLAR-IHC algorithm to evaluate the relative interaction and distance among the different infiltrating cells. Combination of such evaluations together with t-distributed stochastic neighbor embedding and uniform manifold approximation and projection can help to identify prediction markers for a better patients’ stratification for immunotherapy.

From the clinical viewpoint, he first presented their strategy to solve the problem regarding patient stratification for clinical PD1/PD-L1 blockade. Indeed, as previously introduced by A. Hartmann, the different clones used for determination of the PD-L1 status provide distinct staining patterns, with clone SP142 predominantly staining immune cells, while clone 22c3 mainly stains tumor cells. To provide a more solid approach, a staining panel combining three different anti-PD-L1 Ab (SP142, 22C3 and SP263) together with tumor and immune markers, like EpCAM and CD45, respectively, is currently being used to allow a better characterization of PD-L1 expression within the tumor and possibly a better stratification for the iCPI therapy.

With these approaches, he was able to identify different markers in different tumor entities that predict response to PD1 blockade. For example, patients with NSCLC that in the Asian population are linked to EGF-R mutation, have increased response rates to PD1/PD-L1 blockade if they have higher numbers of CD39^+^ CD8^+^ T cells. In contrast, in HCC, that in the Asian population is linked to viral infection like HBV and HCV, better responses correlated with the presence of CD38^+^ CD68^+^ macrophages and their ability to produce pro-inflammatory cytokines, like TNF-α and IL-6. The presence of a CD38^+^ infiltrate was also found to predict resistance to standard therapy with Sorafenib and, therefore, can be used for stratifying patients for alternative therapies.

**Cornelis Melief** (Department of Immunohematology and Blood Transfusion, Leiden University Medical Centre, Leiden, The Netherlands) focused on active immunotherapy in the setting of human papilloma virus (HPV)16 associated tumors. The ISA101 vaccine consists in a pool of synthetic long peptides covering the E6 and E7 proteins of HPV16 that is injected subcutaneously as an emulsion in of the adjuvant montanide ISA-51. In this way, a direct presentation of antigens in tolerogenic conditions is avoided, because the peptides need processing by professional antigen-presenting cells (APCs) like DCs that are also matured by the co-delivered adjuvant so that there is an increased possibility to induce strong immune responses. Moreover, since DCs can process the peptides into HLA class I and class II epitopes, a sustained Th1 and CTL response can be induced.

Whereas this vaccine is effective as monotherapy in a pre-malignant setting, where higher HPV-specific response correlated with clinical outcome, it does not have a therapeutic efficacy in advanced cancer patients despite the fact that it can induce a weak antigen-specific response. To improve the patients’ outcome, combinations with other approaches that can help to overcome the generalized immunosuppression of the cancer patient as well as the hostile TME, were evaluated. On one side, vaccination has been combined with iCPI and in a phase II study in HPV16^+^ HNSCC addition of ISA101 before and during Nivolumab (anti-PD1) treatment doubled the tumor response rate resulting in an ORR of 36%.

In a second setting, vaccination was combined with standard chemotherapy with carboplatin and paclitaxel in patients with cervical cancer. In this setting, the first dose of the peptide vaccine was given after the second of six cycles of chemotherapy, when a strong decrease in myeloid cells in blood PBMC was recorded. Results from the phase I/II trial evaluating escalating doses of the vaccine indicate that the strength of the vaccine-induced HPV-specific immune response correlates with clinical outcome, whereas the general fitness of the immune system evaluated as response to common microbial antigens had no role. Since in the low responder patients enhanced levels of Tregs were found, the plan for the future is to combine vaccination with Treg depleting strategies.

**Stina Wickström** (Cancer Center Karolinska, Stockholm, Sweden) presented results from the MAT02 clinical trial treating metastatic melanoma patients with ACT. The trial was divided into two different cohorts (due to safety reasons), where the patients in cohort (a) received in vitro expanded TILs and IL-2, while cohort (b) received in vitro expanded TIL, IL-2 and a unique DC vaccine program to boost in vivo the anti-tumor immune response. For this purpose, autologous DCs were obtained and pulsed with crude tumor lysate before in vivo injection. This allowed not only partial responses, but also two complete responses among treated patients.

To clarify the question of how long TIL clones remain in the patient and which TCRs are retained, TCR sequencing was performed on the infusion product as well as on blood samples taken before and after infusion. In various patients, some of the clones present in the TIL product expanded in the blood as well as in a biopsy taken at the DC vaccination site and some could still be detected after 2 years.

Using this approach, two problems arise, such as the need of highly infiltrated tumors to be able to expand the TILs and enough tumor material for loading the DCs. Alternative strategies would be the expansion of tumor specific T cells from the peripheral blood and the use of tumor biopsies for sequencing to identify specific neoantigens and predict epitopes binding to the patients’ HLA molecules to be used for DC loading. Preliminary data confirm the possibility of such approach with the expansion of antigen specific T cells capable of recognizing tumor cells in vitro that have been amplified from blood samples also derived from healthy donors.

**Christopher A. Klebanoff** (Center for Cell Engineering, Memorial Sloan Kettering Cancer Center, New York, USA) presented two strategies to improve the efficacy of ACT. In the first setting he demonstrated that expansion of T cells in the presence of high concentrations of potassium ions ([K^+^]), like those found in the TME, cause functional dysfunction in the T cells and induce characteristics of stemness that promote T-cell persistence in vivo. Evaluation of the underlying mechanism indicates that in the presence of high extracellular [K^+^] T cells are unable to acquire glucose from the extracellular space resulting in a metabolic impairment that induce the auto-phagocytic pathway. Moreover, characteristics of stemness like gene expression, such as CXCR5, SELL and TCF7, are induced and the T cells are more proliferating. When transferred into B16 melanoma bearing mice, these T cells improved the rejection of the tumor and increased the survival of mice.

In the second setting, he proposed the transfection of T cells with a dominant negative form of the death receptor Fas to reduce apoptosis of transferred cells. Indeed, during expansion for ACT activated T cells upregulate the Fas receptor. Since the majority of human cancers overexpress its ligand, and it is also found on the vasculature, infiltrating T cells would quickly undergo apoptosis. Experiments performed in the murine in vitro setting indicate that transfected expanded T cells survive upon altered Fas triggering, but also upon cytokine withdrawal. Moreover, transfer experiments using TCR transgenic of chimeric antigen receptor (CAR) T cells indicate enhanced persistence in the spleen, but also within the tumor resulting in tumor regression and enhanced survival of mice. Similar results of enhanced survival of human T cells transfected with a human dominant negative receptor were also presented.

**Sibylle Loibl** (Centre of Haematology and Oncology, University Frankfurt/Main, Germany) gave an update of different trials in metastatic and non-metastatic breast carcinoma implementing iCPI together with chemotherapy or targeted therapy. For example in the Impassion 130, the anti-PD-L1 Ab Atezolizumab was combined with Paclitaxel resulting in an improved outcome for patients with a PD-L1^+^, but not negative tumor staining. Combination of PD1 or PD-L1 blockade with different inhibitors of the DNA damage response, like in the Topacio or the Mediola trials, resulted in a clinical improvement, but the responses were not correlated with the g*BRCA* mutation. To find the best therapeutic approach to be given before PD1 blockade, a “pick the winner” study was performed comparing radiotherapy or chemotherapy with cyclophosphamide, cisplatin or doxorubicin as single agents, with doxorubicin providing the strongest ORR and thus being further evaluated in an expanded cohort.

In the GeparNUEVO study, non-metastatic triple negative breast cancer (TNBC) patients underwent neoadjuvant chemotherapy, consisting of 12 weeks of nab-Paclitaxel followed by 8 weeks of epirubicin and cyclophosphamide, combined with placebo or with the anti-PD-L1 Ab Durvalumab. Compared to placebo, the combination therapy with Durvalumab resulted in an enhanced pathological complete response (pCR) that reached statistical significance in the “Window Cohort” that received two injections of Durvalumab before the start of chemotherapy. Stronger effects were also observed in TNBC patients under 40 years, with state IIA or higher tumors and with high frequencies of stromal TIL.

**Bernard A. Fox** (Knight Cancer Institute, OHSU Portland, Oregon, USA) shifted from blocking iCP molecules to triggering costimulatory receptors, such as OX40. Attempts to trigger agonist receptor on T cells as single agent have not been successful in the clinic, which was probably related to the missing expression of the targeted receptor, when the Ab was injected. Indeed, agonist receptors have a tighter expression pattern, with OX40 that is upregulated for example within 24 h from TCR triggering and then turn to background levels in the following 72 h. Thus, to successfully implement such agents, T cells have to be activated via, e.g., vaccination thus posing the problem of which TCR specificity can be triggered. As a solution, a “wide covering” vaccine that would reduce escape variants and that could be used in many different tumor types was created, namely, the DPV-001 vaccine. It is an “off the shelf” vaccine consisting of a double membrane vesicle that has CLEC9A molecules on the surface for in vivo targeting to DCs, while within there are different adjuvants, such as toll like receptor (TLR) agonists and danger associated molecular patterns (DAMP) for proper DC activation together with more than hundred putative cancer antigens identified using TCGA data as commonly overexpressed shared tumor antigens. This vaccine was implemented in a phase II clinical trial in NSCLC patients and in vitro evaluation indicates that it was able to induce and/or augment a cellular as well as a humoral immune response. Based on these results, a new immunotherapy concept was proposed, where the immune system “engine” is first started with vaccination, gas is then given by triggering costimulatory agonist, such as OX40 or GITR and later the brake is released by blocking iCPI like the PD1/PD-L1 axis. Such strategy is currently applied in phase I clinical trials in TNBC and HNSCC combining DPV-001 with OX40 or GITR, respectively, and then with blockade of the PD1/PD-L1 axis.

**Alessia Covre** (Center for Immuno-Oncology, University Hospital of Siena, Italy) presented an alternative approach for melanoma therapy based on epigenetic remodeling. Three different demethylating agents were compared for their in vitro effects on melanoma cell lines demonstrating a stronger immunomodulatory activity of Guadecitabine, a next-generation DNA hypomethylating agent, and Decitabine, compared to 5′-Azacytidine. These agents were able to induce the upregulation of HLA class I molecules, as well as of different tumor-associated antigens and adhesion molecules, like ICAM-1 that could restore interaction with T cells. On the other side, they also induced an upregulation of CTLA-4, PD1 and PD-L1 that could impair T-cell recognition, supporting the idea to combine epigenetic drugs and iCPIs to improve the therapeutic efficacy of cancer immunotherapy.

Preclinical evaluation of the Guadecitabine treatment in mice with syngeneic mammary carcinoma revealed an upregulation of different immune pathways and enhanced anti-tumor responses when combined with CTLA-4 blockade, thus paving the way for translation into the phase I clinical trial NIBIT-M4. Stage III/IV melanoma patients were treated with three different doses of Guadecitabine in combination with the anti-CTLA4 Ab Ipilimumab and biopsies of the tumors were taken before and along therapy to evaluate the effect of treatment. To sum up, the treatment was safe and well tolerated with only some myelotoxicity at the highest concentration tested. As for the murine setting, Guadecitabine treatment reduced the methylation rate in the tumor of patients with an induction of many immune pathways with respect to pre-treatment samples. They found an ORR of 26% and the comparison of responder and non-responder patients’ highlighted increased induction of many immunological pathways along treatment, but also some pre-existing differences.

Next to the talks in the main symposium, posters and short talks were presented on Thursday by young investigators. These were evaluated by two international speakers, who decided the prizes for the best posters and talks.

The three poster prizes were awarded to:

1st Poster Prize: Liliana Loureiro (Helmholtz-Zentrum Dresden-Rossendorf, Germany)

2nd Poster Prize: Eric Freund (INP Greifswald, Germany)

3rd Poster Prize: Karthikeyan Subbarayan and Maria-Filothei Lazaridou (Martin-Luther-University Halle-Wittenberg, Germany).

The three talk prizes were awarded to:

1st Talk Prize: Bastian Kruse (Universitätsklinikum Magdeburg, Germany)

2nd Talk Prize: Linda Bilonda Mutala (Université de Nantes, France)

3rd Talk Prize: Maximilian Kiessler (Technischen Universität Dresden, Germany).

The special SITC Prize was awarded to Claudia Arndt (Helmholtz-Zentrum Dresden-Rossendorf, Germany).

## Conclusions

The meeting gave an overview of some of the hot topics in the field of tumor immunology and immunotherapy, but also discussed future strategies for optimization of the patients’ outcome. It highlights the role of immunotherapy as a fourth pillar for tumor treatment, but also demonstrates that still an increased knowledge of basic tumor immunology including immune escape mechanisms, molecular gene expression patterns and TMB and the composition of the TME is required to improve and optimize immunotherapy or to develop novel therapeutic strategies for the treatment of patients.

## Abbreviations

Ab: Antibody
ACT: Adoptive cell therapy
ADCC: Antibody-dependent cellular cytotoxicity
APC: Antigen-presenting cell
APM: Antigen-processing machinery
β_2_m: Beta2-microglobulin
bgn: Biglycan
γδ: Gamma/delta
CAR: Chimeric antigen receptor
COX 2: Cyclooxygenase 2
CRC: Colorectal cancer
CTLA-4: Cytotoxic T-lymphocyte-associated protein-4
DC: Dendritic cells
ECM: Extracellular matrix
EGF-R: Epidermal growth factor receptor
EMA: European Medicine Agency
FDA: Food and Drug Administration
GvHD: Graft-versus-host disease
HCC: Hepatocellular carcinoma
HLA: Human leukocyte antigen
HNSCC: Head and neck squamous cell cancer
HPV: Human papilloma virus
ICOS: Inducible T-cell costimulator
iCPI: Immune checkpoint inhibitor
IFN: Interferon
IPP: Isopentenyl-pyrophosphate
k.o.: Knock out
LDH: Lactate dehydrogenase
LPS: Lipopolysaccharide
MDSC: Myeloid-derived suppressor cells
miRNA: MicroRNA
MHC: Major histocompatibility complex
MM: Malignant melanoma
MPE: Molecular pathological epidemiology
MSI: Multispectral imaging
NK: Natural killer
NSCLC: Non-small-cell lung carcinoma
NGS: Next-generation sequencing
NKCE: NK-cell engager
nRCT: Neoadjuvant radio-chemotherapy
ORR: Objective response rate
OS: Overall survival
OSCC: Oral squamous cell carcinoma
PBMC: Peripheral blood mononuclear cells
pCR: Pathological complete response
pDC: Plasmacytoid DC
PD: Programmed death
PD-L1: Programmed death ligand 1
PFS: Progression free survival
PSGL1: P-selectin glycoprotein ligand 1
RCC: Renal cell carcinoma
SIRPα: Signal regulatory protein α
SlanMo: 6-Sulfo LacNAc (slan) modification of PSGL1 expressing monocytes
TAP: Transporter associated with antigen processing
TCGA: The Cancer Genome Atlas
TCR: T-cell receptor
TGF: Transforming growth factor
TIGIT: T-cell immunoglobulin and immune receptor tyrosine-based inhibitory motif
TIL: Tumor-infiltrating lymphocyte
TMB: Tumor mutational burden
TME: Tumor microenvironment
TNBC: Triple negative breast cancer
TNF: Tumor necrosis factor
Treg: Regulatory T cell
UTR: Untranslated region

